# Draft Genome Sequences of Two *Mycobacterium bovis* Strains Isolated from Beef Cattle in Paraguay

**DOI:** 10.1128/genomeA.00616-17

**Published:** 2017-07-13

**Authors:** Lidia Sanabria, Lorena Lagrave, Christiane Nishibe, Augusto C. A. Ribas, Martín J. Zumárraga, Nalvo F. Almeida, Flábio R. Araújo

**Affiliations:** a Servicio Nacional de Calidad y Salud Animal (SENACSA), Asunción, Paraguay; b School of Computing, UFMS, Campo Grande, Mato Grosso do Sul, Brazil; c Instituto de Biotecnología, CICVyA-INTA, Hurlingham, Buenos Aires, Argentina; d Embrapa Beef Cattle, Campo Grande, Mato Grosso do Sul, Brazil

## Abstract

This work reports the draft genome sequences of the *Mycobacterium bovis* strains M1009 and M1010, isolated from the lymph nodes of two infected cows on a beef farm in Paraguay. Comparative genomics between these strains and other regional strains may provide more insights regarding *M. bovis* epidemiology in South America.

## GENOME ANNOUNCEMENT

Bovine tuberculosis (bTB) is an infectious chronic disease caused by *Mycobacterium bovis*. This species exhibits a broad host range encompassing bovine and other domesticated mammals, besides wildlife animals (1) and humans (2). In Paraguay, bTB is a notifiable disease regulated by a national disease eradication program (http://www.senacsa.gov.py/application/files/4414/6158/9486/PRY-DEC-18613-1997.pdf). An update on bTB programs in Latin American and Caribbean countries pointed out a prevalence of 0.7% (0 to 2.8%) in 10 districts of Paraguay (3). Eradication of bTB is an important goal to mitigate the risk of zoonotic transmission, and it also has an impact on international trade (4, 5).

As a strategy to eradicate the disease, it is important to characterize the molecular diversity of the *M. bovis* strains isolated in Paraguay. We sequenced and annotated draft genome sequences of the *M. bovis* M1009 and M1010 strains, isolated from the retropharyngeal, hepatic, mesenteric (M1009), and prescapular (M1010) lymph nodes of two mixed breed cows from a beef farm in Presidente Hayes District. Both strains displayed the SB0267 spoligotype, which is also present in Argentina and Brazil (6) and the United Kingdom (http://www.mbovis.org/database.php).

Genome sequences were obtained using Illumina MiSeq (7), producing 5,439,661 paired-end reads for M1009 and 6,003,419 paired-end reads for M1010. After filtering with PRINSEQ (8), 4,919,772 and 5,423,808 paired-end reads were used in the assemblies of the M1009 and M1010 strains, respectively. We performed reference-assisted genome assembly of filtered data using the *M. bovis* AF2122/97 genome as a reference (GenBank accession no. LT708304.1) and using Bowtie2 (9). The assemblies produced 29 contigs to M1009 and 28 contigs to M1010 (all contigs no shorter than 500 bp), with corresponding coverages of 316.31-fold and 343.06-fold and *N*_50_ contig sizes of 300,837 bp and 306,712 bp. Annotations for M1009 and M1010 were obtained by using NCBI PGAP (10), resulting in 4,025 and 4,026 coding genes (2,886 and 2,888 of them with functional assignment), respectively. Each strain has 133 pseudogenes, 45 tRNAs, 3 rRNAs, and 3 noncoding RNAs (ncRNAs).

Comparative genomics between these strains and other regional ones may provide more insights regarding *M. bovis* epidemiology in Paraguay, notably the patterns of host or spatial associations, the differentiation of lineages and phylogenetic structures among *M. bovis* strains, and the persistence of particular genotypes in a region (11, 12).

### Accession number(s).

The draft genome sequences of *M. bovis* strains M1009 and M1010 have been deposited at DDBJ/EMBL/GenBank with accession numbers NBZZ00000000 and NCTD00000000, respectively, both in their first versions, and under BioProject PRJNA214551.
